# Biological Instability in a Chlorinated Drinking Water Distribution Network

**DOI:** 10.1371/journal.pone.0096354

**Published:** 2014-05-05

**Authors:** Alina Nescerecka, Janis Rubulis, Marius Vital, Talis Juhna, Frederik Hammes

**Affiliations:** 1 Department of Water Engineering and Technology, Riga Technical University, Riga, Latvia; 2 Department of Environmental Microbiology, Eawag, Swiss Federal Institute for Aquatic Science and Technology, Dübendorf, Switzerland; Catalan Institute for Water Research (ICRA), Spain

## Abstract

The purpose of a drinking water distribution system is to deliver drinking water to the consumer, preferably with the same quality as when it left the treatment plant. In this context, the maintenance of good microbiological quality is often referred to as biological stability, and the addition of sufficient chlorine residuals is regarded as one way to achieve this. The full-scale drinking water distribution system of Riga (Latvia) was investigated with respect to biological stability in chlorinated drinking water. Flow cytometric (FCM) intact cell concentrations, intracellular adenosine tri-phosphate (ATP), heterotrophic plate counts and residual chlorine measurements were performed to evaluate the drinking water quality and stability at 49 sampling points throughout the distribution network. Cell viability methods were compared and the importance of extracellular ATP measurements was examined as well. FCM intact cell concentrations varied from 5×10^3^ cells mL^−1^ to 4.66×10^5^ cells mL^−1^ in the network. While this parameter did not exceed 2.1×10^4^ cells mL^−1^ in the effluent from any water treatment plant, 50% of all the network samples contained more than 1.06×10^5^ cells mL^−1^. This indisputably demonstrates biological instability in this particular drinking water distribution system, which was ascribed to a loss of disinfectant residuals and concomitant bacterial growth. The study highlights the potential of using cultivation-independent methods for the assessment of chlorinated water samples. In addition, it underlines the complexity of full-scale drinking water distribution systems, and the resulting challenges to establish the causes of biological instability.

## Introduction

The goal of public drinking water supply systems is to produce water of acceptable aesthetic and hygienic quality and to maintain that quality throughout distribution until the point of consumption. From a microbiological perspective, the quality of treated water can deteriorate as a result of excessive bacterial growth, which can lead to problems such as a sensory deterioration of water quality (e.g. taste, odor, turbidity, discoloration) as well as pathogen proliferation [1]–[10]. To avoid this, biological stability during distribution can be achieved by maintaining sufficient residual disinfectants in the water, and/or through nutrient limitations [3], [7], [11], [12]. However, drinking water systems should not be viewed as sterile; complex indigenous bacterial communities have been shown to inhabit both chlorinated and non-chlorinated drinking water distribution systems [5], [13]–[17].

**Figure 1:**
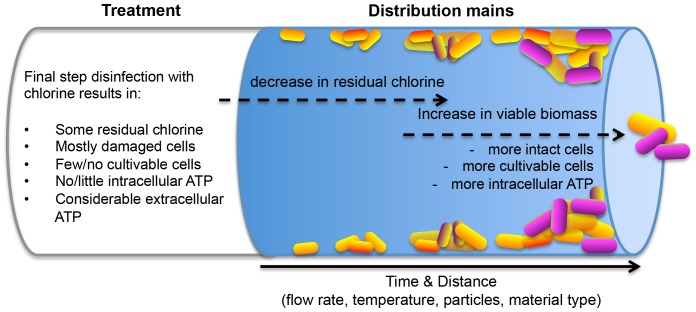
A worst-case-scenario in an unstable, chlorinated distribution network. Prediction of changes in the microbiological state of the water due to the depletion of residual chlorine and the concomitant growth of bacteria, potentially resulting in hygienic and sensory deterioration of the water quality.

The concept of biological stability and its impact on a system's microbiology has been discussed extensively in the framework of non-chlorinated drinking water distribution systems [3], [7], [17]–[20]. However, many treatment plants worldwide employ a final disinfection step to ensure that no viable bacteria enter the distribution system. The latter is often achieved by oxidative disinfection, usually by chlorination [21]. Disinfection has a number of implications for a biological system. During chlorination, one can expect that a considerable fraction of bacteria in the water are killed or damaged, while some residual chlorine may remain in the water (Figure 1). This could be visible through numerous microbial monitoring methods. For example, the number of cultivable bacteria, measured with heterotrophic plate counts, would reduce dramatically [22], [23]. Secondly, bacteria cells are likely to display measurable membrane damage irrespective of their cultivability [24], though the rate and extent of damage may differ between different communities. This would be detectable with several staining techniques coupled with epifluorescence microscopy or flow cytometry (FCM). Also, adenosine tri-phosphate (ATP), often used as a cultivation-independent viability method [19], [22], [25] will be severely affected. Based on data from Hammes and co-workers [4] one may reasonably expect increased levels of extracellular ATP (so-called free ATP) and decreased concentrations of intracellular ATP (bacterial ATP) following oxidative disinfection. Irrespective of the detection method, the overall consequence of disinfection is a considerable decrease in the viable biomass, potentially opening a niche for microorganisms to occupy downstream of the treatment process. Following initial disinfection, residual chlorine might provoke undesirable changes during drinking water distribution. Disinfectants target not only bacteria, but it also react with natural organic matter, pipe surfaces and particles in the network, thus potentially forming/releasing assimilable organic carbon (AOC) [26]–[30]. AOC can easily be consumed by bacteria, and is therefore seen as a main contributor to biological instability. Moreover, chlorine decay within the network negatively affects its ability to inhibit microbial growth at the far ends of the network [12]. If all factors were considered, the presence of nutrients, a reduction in the number of competing bacteria, and the lack of residual disinfectant would potentially lead to biological instability in the distribution network, manifesting in a subsequent bacterial growth (Figure 1). Besides the importance of nutrients, the extent of bacterial growth will be influenced by a number of factors. For example, increased water temperature can accelerate chlorine decay and favor bacteria growth [19], [31], while changes in hydraulic conditions can alter nutrient supply for microorganisms in biofilms and/or bacteria detachment from the pipe surfaces [32], [33]. Finally, the quality of materials in contact with drinking water, as well as the presence of sediments and loose deposits, can both affect the general microbial quality of the water [6], [34], [35].

In the present study we examined some of the above-discussed concepts in a full-scale, chlorinated distribution system in the city of Riga (Latvia) with a number of microbiological methods. The purpose was a detailed investigation of the entire city's distribution network, asking the basic question whether evidence of spatial and/or temporal biological instability exists, and if so, to which degree. Additional goals were to evaluate the use of fluorescent staining coupled with FCM, as well as ATP analysis, for the assessment of chlorinated drinking water in a distribution network with disinfectant residuals.

## Materials and Methods

### Ethics statement

Permission for sampling at all locations in the present study was obtained from the local water utility (Rīgas 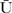
dens).

### Description of study site

Sampling was performed in the full-scale distribution network of Riga (Latvia) with a total length of about 1400 km. The city is supplied with drinking water from six water treatment plants (WTP) produced from both surface and groundwater (150 000 m^3^ d^−1^). Only the three major WTP, which are continuously operated, were included in the sampling campaign. Average WTP effluent water quality parameters for each treatment plant are shown in Table 1. The distribution network mainly consists of cast iron (80%) and unlined iron (15%) pipes as old as 50 years. The diameters of pipes ranged from 100 to 1200 mm. Three reservoirs are operated in the network to compensate for fluctuations in the daily water demand, while four high-pressure zones are maintained in some distal areas of the network. The high-pressure zones were excluded from the present study. A total of 49 sampling sites were selected across the city to cover the network broadly and to include both proximal and distal zones relative to the treatment plants. The sampling sites were selected according to the approximate water retention times obtained from a validated hydraulic model made in EPANET 2.0 [36], [37] based on a total length of 538 km (39% of the total length of the network). Apart from the effluents of the three treatment plants, the sampling sites were in all cases fire hydrants in order to attain some degree of reproducibility between sampling and to avoid localized effects (e.g. household growth). The exact locations of sampled fire hydrants can be obtained from the authors after agreement from the local water utility.

**Table 1:** Average water quality parameters for the final effluents of the the three main treatment plants of Riga (Latvia).

	WTP 1	WTP 2	WTP 3
**Source water**	surface water	artificially recharged groundwater	groundwater
**Final treatment step** a	Cl_2_ (0.5–3 mg L^−1^)	Cl_2_ (ca. 1.5 mg L^−1^)	N.A.
**Residual chlorine (mg L^−1^)** b	0.44±0.11	0.51±0.01	0.42±0.26
**Total organic carbon (TOC) (mg L^−1^)** a	6±1	9±3	3
**Assimilable organic carbon (AOC) (µg L^−1^)** a	213±37	209±59	N.A.
**Total cell concentration (cells mL^−1^)** b	5.31±0.97×10^5^	5.45±0.47×10^5^	1.69±0.18×10^5^
**Intact cell concentration (cells mL^−1^)** b	1.83±1.18×10^4^	1.4±0.86×10^4^	1.03±0.68×10^4^
**Total ATP (nM)** b	0.015±0.005	0.029±0.004	0.011±0.002
**Intracellular ATP (nM)** b	0.007±0.003	0.000±0.004	0.001±0.002
**HPC 22°C (CFU mL^−1^)** b	23±24	4±2	4
**HPC 36°C (CFU mL^−1^)** b	16±16	4±2	1
**Conductivity (µS cm^−1^), 25°C** a	468±101	625±4	272±25
**pH** a	6.63±0.18	7.41±0.04	7.5±0.05

a Data supplied by the water utility or measured in previous sampling campaigns.

b Data from present study.

### Sampling protocol

**Figure 2:**
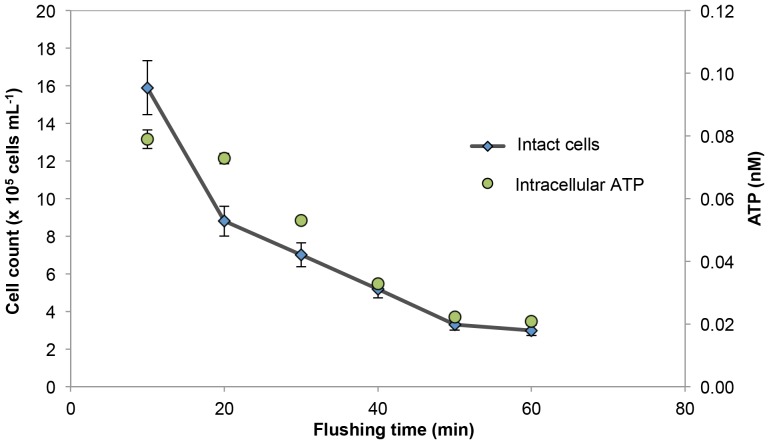
The impact of low velocity flushing on the water quality in a newly-opened fire hydrant. Intracellular adenosine tri-phosphate (ATP) data points were derived from duplicate measurements of extracellular and total ATP concentrations. FCM intact cells (after SYBR Green I and propidium iodide staining) were single measurements, with a relative standard deviation of 9% calculated from all data in the present study.

A specific sampling protocol was designed and followed in order to avoid artifacts due to water stagnation in unused fire hydrants. Each hydrant was pre-flushed at a high velocity (never exceeding 1.6 m s^−1^) for no more than 60 s, then immediately adjusted to a low velocity of 0.015–0.25 m s^−1^ and connected to an online system for monitoring pH, temperature, redox potential, electro-conductivity and turbidity. The low sampling velocity was specifically used to ensure a minimal possible impact of cell wall erosion and detachment from biofilms on the samples and measurements. Readings of all parameters were taken at 5–10 minute intervals, and water was only sampled for microbiological analysis once all of the parameters stabilized. The impact of this hydrant flushing is demonstrated in an example in Figure 2 and discussed in detail in the results section. Samples were kept in cold storage (≈5°C) and analyzed within four hours of sampling.

### Chemical analysis

Determination of free chlorine was performed according to standard method EN ISO 7393-1, based on the direct reaction with N,N-diethyl-1,4-phenylenediamine (DPD) and subsequent formation of a red compound at pH 6.2–6.5. Afterwards titration by means of a standard solution of ammonium iron (III) sulfate until disappearance of the red color was performed. Determination of total chlorine was performed according to EN ISO 7393-1, based on the reaction with DPD in the presence of an excess of potassium iodide, and then titration as described above.

### Fluorescent staining and flow cytometry (FCM) of water samples

Staining and FCM analysis was done as described previously [4], [38]. In short, for a working solution, SYBR^®^ Green I (SG) stock (Invitrogen AG, Basel, Switzerland) was diluted 100x in anhydrous dimethylsulfoxide (DMSO) and propidium iodide (PI; 30 mM) was mixed with the SYBR^®^ Green I working solution to a final PI concentration of 0.6 mM. This working solution was stored at −20°C until use. From every water sample, 1 mL was stained with SGPI at 10 µL mL^−1^. Before analysis, samples were incubated in the dark for 15 minutes. Prior to FCM analysis, the water samples were diluted with 0.22 µm filtered bottled water (Evian, France) to 10% v/v of the initial concentration. FCM was performed using a Partec CyFlow SL instrument (Partec GmbH, Münster, Germany), equipped with a blue 25 mW solid state laser emitting light at a fixed wavelength of 488 nm. Green fluorescence was collected at 520±10 nm, red fluorescence above 630 nm, and high angle sideward scatter (SSC) at 488 nm. The trigger was set on the green fluorescence channel and data were acquired on two-parameter density plots while no compensation was used for any of the measurements. The CyFlow SL instrument is equipped with volumetric counting hardware and has an experimentally determined quantification limit of 1000 cells mL^−1^ [4].

### Adenosine tri-phosphate (ATP) analysis

Total ATP was determined using the BacTiter-Glo reagent (Promega Corporation, Madison, WI, USA) and a luminometer (Glomax, Turner Biosystems, Sunnyvale, CA, USA) as described elsewhere [25]. A water sample (500 µl) and the ATP reagent (50 µl) were warmed to 38°C simultaneously in separate sterile Eppendorf tubes. The sample and the reagent were then combined and then the luminescence was measured after 20 s reaction time at 38°C. The data were collected as relative light units (RLU) and converted to ATP (nM) by means of a calibration curve made with a known ATP standard (Promega). For extracellular ATP analysis, each sample was filtered through a 0.1 µm sterile syringe filter (Millex-GP, Millipore, Billerica, MA, USA), followed by analysis as described above. The intracellular ATP was calculated by subtracting the extracellular ATP from the total ATP for each individual sample. ATP was measured in duplicate for all samples.

### Heterotrophic plate counts

To obtain heterotrophic plate counts (HPC), samples were serially diluted in sterile distilled water and then inoculated onto nutrient yeast agar plates using the spread plate technique. All plates were incubated in dark at 22°C or 36°C for 3 and 7 days, respectively. Results were expressed as colony forming units (CFU) per ml of water sample.

### Statistical analysis

Statistical data evaluation was performed with the MS Excel Data Analysis tool (Descriptive statistics, Regression). The reproducibility for indirect/calculated data (e.g., intracellular ATP) was calculated by a propagation-of-uncertainty method. FCM data was not always measured in duplicate, due to practical constraints. In these cases, a 9% error (average coefficient of variation (CV) (n = 39)) was applied for representing FCM data. The residual chlorine concentration distribution box plot was created using on-line calculator on http://www.physics.csbsju.edu/stats/.

## Results and Discussion

### The importance of correct sampling

Sample collection during this study elucidated some of the problems specific for this network and highlighted the broader importance of correct sampling procedures. Fire hydrants were selected as sampling points to enable direct access to the distribution network and avoid potential household effects [39]. We opted for a low velocity water flow in combination with online monitoring to achieve comparable samples. In some cases, the water initially emerging from the fire hydrants were visibly turbid and/or discolored (data not shown). Turbid water is clearly unwanted and serves as a first visual confirmation of some form of system failure. In this regard, a recent study in the Netherlands has established an important link between suspended solids and microbial growth and biological instability [6]. Hence in some instances continuous low velocity flushing of up to 60 minutes was required before stable values for chemical and physical parameters as well as microbiological parameters were obtained (Figure 2; Table S1). The data in Figure 2 demonstrate clearly the need for a carefully planned sampling protocol when assessing full-scale systems. It should be noted that Figure 2 represents an example of some of the worst sampling points in the system. Data from other hydrants often showed less fluctuation during flushing (Figure S1). One potential problem during the sampling procedure is the re-suspension of sediments/particles and sloughing of biofilms from the pipes, causing artifacts in the measurements. In this respect, we specifically employed a low velocity (0.015–0.25 m s^−1^) pre-sampling flushing procedure. The latter differs from extreme flushing applied for network cleaning, which is operated with high velocities of 1.5–1.8 m s^−1^ [40], [41]. According to Antoun and co-workers [40] low-veocity flushing (below 0.3 m s^−1^) does not cause any scouring actions. However, it should be considered that part of the samples, especially during the first minutes of the flushing, can cointain biofilm bacteria detatched in a result of pre-flushing [35].

### The concept of detecting instability: a single point in the distribution network

**Figure 3:**
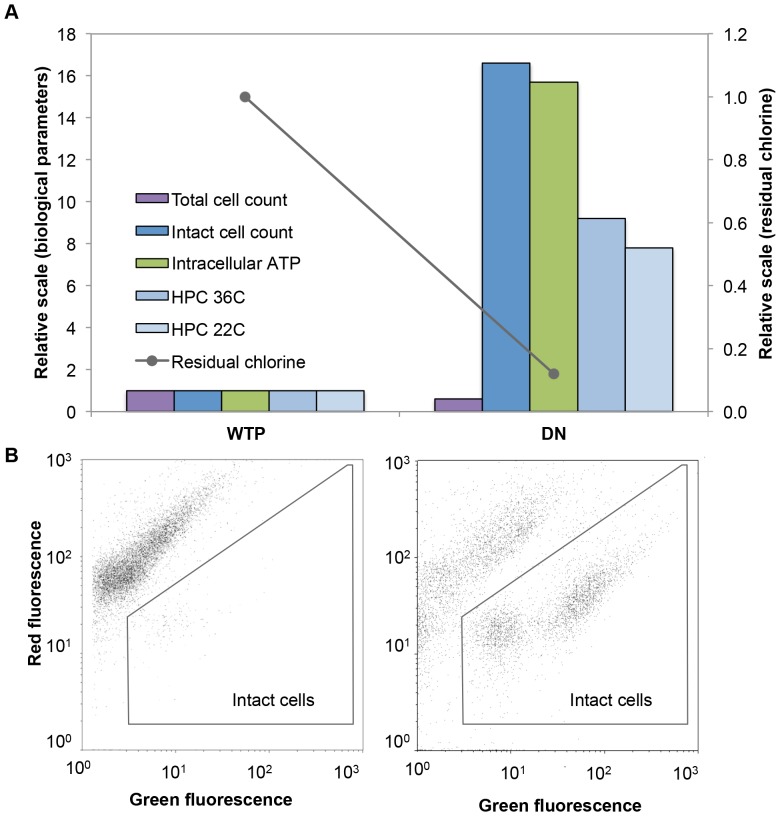
Changes in bacterial parameters between water treatment plant (WTP) and distribution network (DN) sampling points. (A) For comparison, all values at the WTP were set to 1, and values in the DN were expressed relative to their values at the WTP. The original raw data for these samples are shown in Figure S2. Data points are average values for duplicate FCM and ATP measurements and triplicate HPC measurements; (B) Flow cytometric density plots of samples stained with SYBR Green I and propidium iodide, showing the intact cell concentration at the plant and in the specific network point.

In the introduction we proposed the straightforward hypothesis that biological parameters would show an increase between the point of treatment and a point during distribution in case of biological instability (Figure 1). Before the relation between different parameters and the impact on the entire network are discussed in detail below, a single sampling point is compared to its source water as an example to illustrate the concept (Figure 3A). The point was selected on the basis of (1) hydraulic data linking it with a specific WTP, (2) its medial distance from WTP (neither too close and nor too far from the WTP) and, (3) the fact that all microbiological parameters (FCM, ATP and HPC) as well as residual chlorine measurements were performed on this sample. For the purpose of clarity, the data was normalized to the values of the treated water and expressed as the relative change (the raw data and standard deviations for the data in Figure 3A are shown in Figure S2). Evidently the data from Figure 3A supports the basic hypothesis. The microbial parameters such as intact cell concentration, ATP and colony forming units all show a considerable increase in their values. Simultaneously, only 12% (0.06 mg L^−1^) of the initial residual chlorine concentration (0.5 mg L^−1^) was left in the water sample. The data suggests that the residual chlorine in the network was not sufficient to inhibit microbial growth, concurring with earlier report from Prévost and colleagues [42] showing increased HPC, total direct and direct viable bacteria counts in a distribution network coinciding with chlorine depletion. Other studies also showed the presence of viable bacteria in water with chlorine concentration lower than 0.1 mg L^−1^ [23] and that residual chlorine levels below 0.07 mg L^−1^ allows bacterial growth [12]. Data of residual chlorine concentrations in the drinking water network is summarized in Figure S3. Evidently a considerable fraction of samples (18%) had residual chlorine concentrations below 0.1 mg L^−1^.

Staining of bacteria with fluorescent dyes was previously suggested as a way to distinguish between viable and damaged bacteria in real water samples [43], [44], and the application of this approach has been successfully demonstrated in laboratory scale chlorination studies [24], [45]. One focus point of the present study was to determine whether FCM combined with viability staining can be used for a fast and meaningful assessment of viable bacteria in chlorinated drinking water systems. The same samples from Figure 3A, stained with SYBR Green and propidium iodide (SGPI), are shown as density plots obtained with FCM (Figure 3B). The theory behind the staining method and the interpretation of such data are discussed in detail elsewhere [20], [24], [38], [43], [46]. In the treatment plant sample, where the water was recently exposed to chlorine, 98% of all cells were measured as membrane compromised, seen by absence of events inside the gated area of the plot (Figure 3B). In the distribution network (DN) sample, a high concentration of intact cells appeared (Figure 3B). Since these intact cells were clearly not present in the influent, the plausible conclusion is that the bacterial growth occurred during distribution.

### Detailed assessment of dynamic changes in a single point

**Figure 4:**
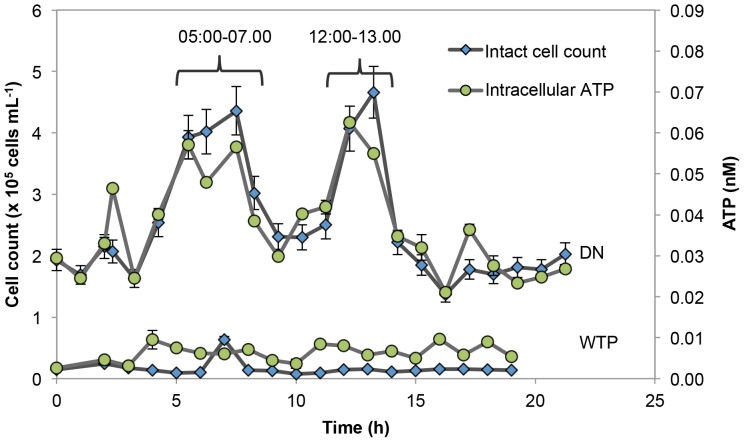
Diurnal changes in bacterial parameters of WTP and DN points. Intensive sampling of one WTP (n = 19) and one point in the DN (n = 23) during 21 hours reveals steady cell concentrations at the treatment plant but clear variations in the distribution network. Intracellular adenosine tri-phosphate (ATP) data points were derived from duplicate measurements of extracellular and total ATP concentrations. FCM intact cells (after SYBR Green I and propidium iodide staining) were single measurements, with a relative standard deviation of 9% calculated from all data in the present study.

High frequency monitoring of a single sampling point revealed temporal instability in the distribution network. We monitored the effluent of one treatment plant and one point in the network with 1-hour intervals during a day (ca. 21 h). The sampling was arranged in such a way that the network sampling started 15 hours after the treatment plant sampling, which corresponded with the estimated water residence time (WRT) for this location. Figure 4 displays the changes of intracellular ATP and intact cell concentrations in the network and the water treatment plant. Values for both parameters were low in the water samples from the treatment plant (n = 19): intracellular ATP varied from 0.0025 nM to 0.0096 nM (mean = 0.0061±0.002 nM) and the intact cell concentration amongst 19 samples varied from 7.5×10^3^ to 6.3×10^4^ cells mL^−1^ (mean = 1.6×10^4^±1.2×10^4^ cells mL^−1^ in average). In turn, the values from the distribution network point (n = 23) were significantly higher: intact cell concentrations ranged from 1.37×10^5^ to 4.66×10^5^ cells mL^−1^ (mean = 2.5×10^5^±9.9×10^4^ cells mL^−1^), and the ATP concentrations from 0.021 to 0.063 nM (mean = 0.038±0.012 nM). Moreover, a distinct pattern was apparent in the distribution network data, with values peaking at about 05:00–07:00 and again at 12:00–13:00. During both these events, the intracellular ATP data followed a similar pattern as the intact cell concentration data, with a good overall correlation (R^2^ = 0.81; p<0.005). Although it is not evident exactly why the bacterial concentrations peaked at these specific time periods, a plausible explanation is a change in the flow velocity due to diurnal changes in water consumption by both industrial and domestic consumers. It was previously shown in laboratory scale experiments that increased flow velocity could lead to increased bacterial detachment from biofilms and a re-suspension of loose deposits, thus leading to an increase in suspended cell concentrations [32], [33], [47]. In addition, it is possible that lower water consumption overnight resulted in considerably reduced flow rates, and consequently a faster decay of chlorine and increased bacterial growth [42], [48].

Detailed data sets of diurnal changes in the microbial quality of water mains, such as Figure 4, are particularly scarce in literature. Importantly, this clearly demonstrated temporal instability in the network for which the exact cause remains uncertain. Moreover, it shows that the absolute cell concentrations at any sampling point may be influenced by the time of sampling.

### Instability data for the entire network

**Figure 5:**
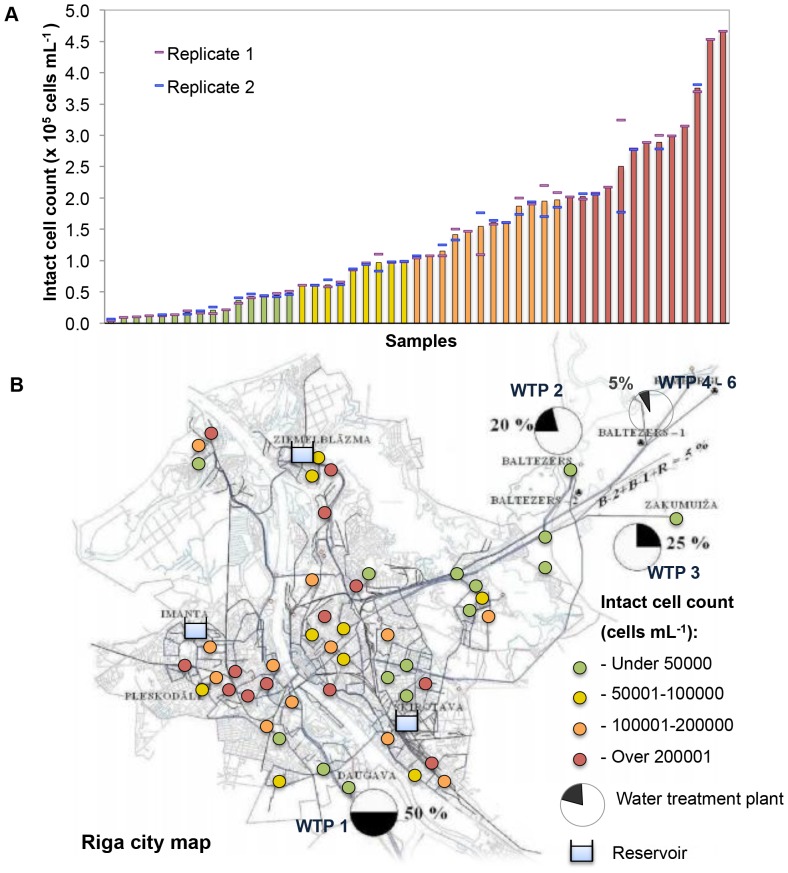
Intact cell concentrations of all samples measured from the distribution network (n = 49). (A) Intact cell concentrations arranged in ascending order and categorized into four main classes (colored bars) according to increasing concentrations. Data points are average values of duplicate measurements. Blue and purple stripes above and below data bars show the measured values. (B) Actual distribution of the classes of intact cells (colored circles) throughout the drinking water distribution network. WTP 1, WTP 2, WTP 3 represent location and productivity of the main water treatment plans supplying the city: WTP 1 operates using surface water, WTP 2 – artificially recharged ground water, WTP 3 – natural groundwater. WTP 4 – 6 indicates on other three pump stations with less significance for the city water supply.

Full-scale distribution networks are complicated systems, not restricted to a single source or a straight distribution line [17]. The Riga distribution network is supplied with drinking water from several separate treatment plants (Table 1). One plant treats surface water from the Daugava River (WTP 1) and the others supply natural groundwater (WTP 3) and artificially recharged groundwater (WTP 2). Chlorination is applied as the final disinfection step at all plants, resulting in low concentrations of intact cells, intracellular ATP and cultivable bacteria in the effluents (Table 1). A large fraction of the active chorine is rapidly consumed due to relatively high levels of organic matter. Despite the fact that the purpose of chlorination and residual chlorine is to limit microbial growth during distribution, a considerable increase in the concentration of intact cells was detected throughout the distribution network. Figure 5A shows the range of intact cell concentrations arranged in ascending order. Treated water contained between 1.84×10^5^–5.63×10^5^ total cells mL^−1^ and between 9.7×10^3^–2.13×10^4^ intact cells mL^−1^ (hence 2–5% intact cells) depending on WTP. The data confirms effective final disinfection in all treatment plants. The total cell concentration values of the drinking water samples from the distribution network (n = 49) varied from 1.62×10^5^ cells mL^−1^ to 1.07×10^6^ cells mL^−1^ and the range of the intact cell concentration was from 5.28×10^3^ cells mL^−1^ to 4.66×10^5^ cells mL^−1^ (3–59% intact cells). Notably, 50% of all samples contained more than 1.06×10^5^ intact cells mL^−1^ corresponding to an increase of at least one order of magnitude in those samples compared to effluent water, which clearly shows that bacterial growth in the distribution network was not an isolated occurrence. The observed increase in intact cell concentration is likely related to the presence of assimilable organic carbon (AOC) in the distributed water. While AOC was not measured in the present study, previous data for two of the treatment plants were high (in the range of 200 µg L^−1^; Table 1), and nutrient availability in the water is generally regarded as a key factor that promotes microbial growth [29], [49]. It cannot be excluded that some variability in the data resulted from bacteria detached from biofilms or re-suspended from sediments during the fire hydrant sampling procedure. However, the potential adverse impact of this was minimized by the low velocity sampling protocol (see above), while the systematic increase in cell concentrations in the network clearly suggests the occurrence of biological instability rather than sampling artifacts. In contrast to these findings, several studies analyzing drinking water distribution systems without any additional residual disinfectants showed no (or only minute) changes in bacterial parameters during distribution [17], [19], [20]. These distributions systems rely on nutrient limitation to achieve biological stability, and while intact cell concentrations are often relatively high (ca. 1×10^5^ cells mL^−1^) [17], [20], changes during distribution tend to be negligible.

To examine the spatial distribution of the growth/instability in the network, the data was divided into four broad categories based on the extent of growth (Figure 5A). These were visualized on the sampling map (Figure 5B). The sampling points with the lowest intact cell concentration (less than 5×10^4^ cells mL^−1^) are marked with green bullets. Yellow and orange colored bullets indicate higher concentrations, while the points with the highest values (over 2×10^5^ cells mL^−1^) are shown as red bullets. As could be expected, the map shows that the points with the lowest cell concentrations are mostly concentrated in areas close to the water treatment plants. Low intact cell concentrations in those areas could be ascribed to (1) disinfection during treatment and (2) growth inhibition from sufficient residual chlorine. Also the flow rate in the outgoing pipes closest to the treatment plants is high, which likely prevents water stagnation, sedimentation and cell adhesion on the pipe surface, and, consequently, biofilm formation and further bacterial growth. A different situation is observed in the distant areas from the water treatment plants and particularly in the so-called mixing zones, where the water from three different water treatment plants potentially mix. The map displays different color points spread in these zones without any visible order. The prevalence of the samples with higher cell concentrations there compared to the areas close to WTPs also corroborates the argument that increasing distance and water residence time could lead to chlorine decay with concomitant oxidation of dissolved organic matter; both these events would favor bacterial growth. Moreover, mixing zones are potential hot-spots for bacterial growth, as one water might well contain the nutrients that are growth limiting in the other.

The uneven spatial distribution of the samples with different intact cell concentrations is noteworthy, highlighted for example by the three points in upper-left corner of the map. Based on the long distance from the WTPs, high intact cell concentrations were expected, but the samples taken from the hydrants located in this small area rather show variability (respectively 1.82×10^4^, 1.87×10^5^, 2.51×10^5^ intact cells mL^−1^). Such different intact cell concentrations could be due to several reasons: the time the samples were taken, which is linked to water consumption and the potential impact of which is shown in Figure 4, the condition of the pipes in this specific area (unknown), the way water flows from the treatment plant, and/or the relative proximity of these sample points to one of the reservoirs (Figure 5B), etc. Other authors showed a decrease in AOC [13] and ATP [19] in the some distal points of the distribution networks. Decrease of AOC concentration was explained by its consumption by bacteria within the network. These authors argued that an insufficient amount of nutrients led to starvation and a decrease in bacterial parameters at the end of the pipelines. However, it is an unlikely reason in the present study, because this phenomenon seems more occasional than systematic.

The combined data demonstrates clearly biological instability throughout the distribution network. However, despite the relative simplicity of the concept (Figure 1; Figure 3A), a complex interplay of chemical, physical and biological parameters and hydraulic conditions should be taken into account for characterization of each particular case of instability.

### Comparison of different microbiological parameters

**Figure 6:**
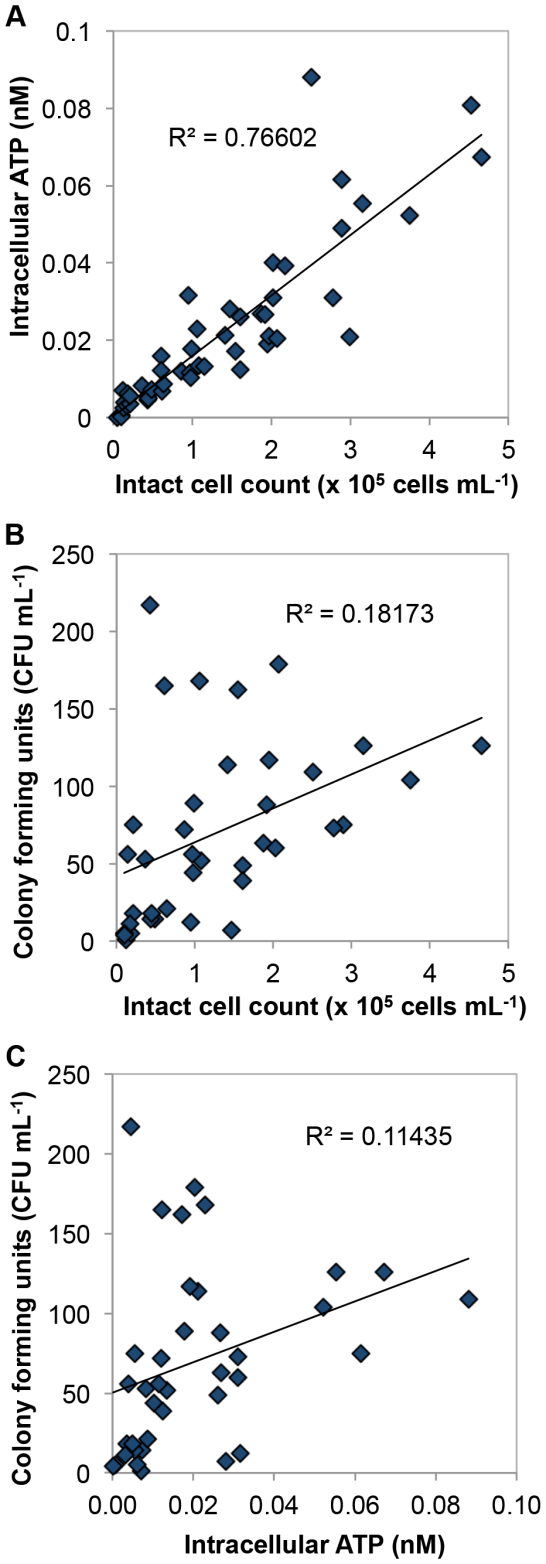
Comparison of the various microbiological parameters. Clear correlations were observed between intact cells and intracellular ATP (n = 49) (A), but no obvious correlations between these two parameters and heterotrophic plate counts at 22°C (n = 38) (B) (C).

FCM and ATP data showed clear correlations, but these data did not correlate well with conventional HPC data. A total amount of 49 different samples was measured in duplicate with ATP (total and extracellular) and FCM (total and intact cell concentration) analyses, while 38 of those samples were further analyzed with HPC. The significant linear correlation (R^2^ = 0.77; n = 49) between intracellular ATP and FCM intact cell concentration is shown in Figure 6A. This corroborates previous studies that showed good results comparing total ATP with total cell concentration [19], [50] and intracellular ATP with intact cell count as well [20], [25]. The strong correlation is encouraging, since FCM and ATP analysis are independent viability parameters – integrity of the cell membrane (FCM) and cellular energy (ATP). A correlation between these parameters during disinfection is not necessarily a given fact. The membrane integrity based PI staining method implies that PI positive cells are damaged and thus considered as inactive, yet extreme examples where living cells became permeable for propidium iodide have been described [46]. In turn, Nocker and co-workers [51] showed that after UV-C exposure cells became inactivated, while their membranes remained essentially intact. Discrepancies between intracellular ATP and intact cell concentration can also result from cell morphology, bacterial species and physiological state, that was discussed in detail previously [25]. The results provided by FCM provide information on single cell level, whereas during ATP analyses the values are evaluated per volume. Hence, intracellular ATP-per-cell was calculated for characterization of biomass activity. In the present study intracellular ATP-per-cell ranges from zero (no cell-bound ATP observed) to 5.92×10^−10^ nM cell^−1^ ( = 3×10^−17^ g cell^−1^) with the average value of 1.68×10^−10^ nM cell^−1^ ( = 8.52×10^−18^ g cell^−1^) (stdev = 9.58×10^−11^ nM cell^−1^, n = 49). The result is in the same range as ATP-per-cell values obtained from various water sources, which were analyzed with the same methods [20], [25]. This suggests that bacterial activity (ATP values) in the intact cells was not affected by any remaining chlorine residuals, and that membrane damage (SGPI values) was in this case reflective of viability in the sample. The good correlation between these two independent parameters is an optimistic prospect for applying these methods for chlorinated water analyses in future studies.

The conventional HPC results were compared with the FCM intact cell concentration values. A weak correlation (R^2^ = 0.18, n = 38) was observed between HPC (at 22°C) and intact cell concentrations (Figure 6B), similar to reports in previous studies [38], [50]. It could be explained by the often described phenomenon, that less than 1% of drinking water bacteria are cultivable on conventional agar plates [25], [50], [52]. In addition, Mezule and co-workers [53] demonstrated evidence of the presence of so called viable-but-not-cultivable (VNBC) bacterial state, in both drinking water and biofilms for the network investigated here, thus indicating further limitations in the HPC method. Since intracellular ATP showed a good correlation with intact cell concentration, but intact cell count correlated weakly with HPC, it was expected that intracellular ATP and HPC would not correlate well (Figure 6C; R^2^ = 0.11, n = 38). Various studies were performed to compare ATP and HPC parameters from water samples, but good correlations were never observed e.g., R^2^ = 0.20 [19], R^2^ = 0.36 [22] and R^2^ = 0.31 [50]. Our results combined with those from previous studies cast further doubts on the value of using the HPC method for general microbiological drinking water quality control. In our opinion, the clear correlation between two methodologically independent viability parameters (intracellular ATP and FCM intact cell counts), and the absence of any correlations with two different HPC methods, renders the former methods more meaningful for assessing and understanding biological instability, particularly in chlorinated environments.

### Importance of measuring extracellular ATP

**Figure 7:**
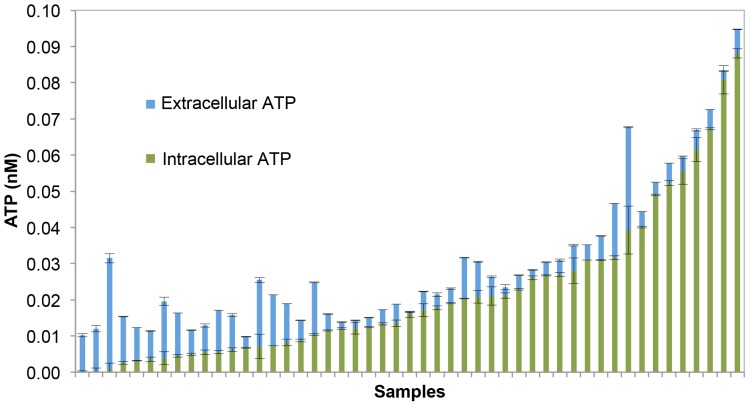
Distribution of intracellular and extracellular ATP in the water samples. In general, higher concentrations and relative percentages of extracellular ATP were measured in samples that exhibited lower intracellular ATP concentrations (n = 49).

Arguments for and against the concept and importance of measuring extracellular ATP have been made [4], [19], [20], [25], [54], [55]. To understand this better, we arranged our data according to increasing intracellular ATP concentrations, after which the measured extracellular ATP values were added to each corresponding sample (Figure 7). It is evident that extracellular ATP constitutes a considerable fraction of the total ATP amount in some samples – varying from 3% up to 100% – with an average contribution of 36% (n = 49). Moreover, 33% of the samples contain more that 50% of extracellular ATP. This data supports other studies, where analyses showed high extracellular ATP in drinking water samples from the distribution networks [20], [25]. Interestingly, the highest extracellular ATP ratio is mostly observed in the samples with relatively low intracellular ATP, in this case samples with close proximity to the treatment plant. In the case of chlorinated water, this could potentially be explained by the oxidative effect of chlorine on bacterial cells. Previous studies have shown extensive damage to bacterial membranes during chlorination [24], [45], after which a release of extracellular ATP from the damaged bacteria can occur. This membrane damage was also clearly detected in the present study (e.g., Figure 3B). Although, there is lack of detailed data considering the release of extracellular ATP in water samples affected by chlorination, strong evidence of ATP release during oxidation was presented in previous studies [4], [20]. Both these works showed a significant decrease in cell concentrations and intracellular ATP after ozonation, whereas extracellular ATP comprised 83–100% of the total ATP. Moreover, Figure 7 shows that samples with increased intracellular ATP concentrations, which we linked to bacterial growth during distribution, often had considerably less extracellular ATP in relation to total ATP. This could be due to the fact that extracellular ATP can be biodegraded by bacteria or extracellular enzymes in the network [54], [56]–[58]. However, it cannot be excluded that a decrease in extracellular ATP during distribution occurs due to oxidation by residual chlorine present in the network.

## Conclusions

- An investigation of a full-scale chlorinated drinking water distribution network with various microbiological methods clearly demonstrated both spatial and temporal biological instability in the network.
- Fluorescent staining with SGPI in combination with ATP measurements provided reliable and descriptive information about bacterial density and viability in chlorinated drinking water samples.
- A good correlation was observed between intracellular ATP and intact cell counts (R^2^ = 0.77), whereas HPC showed poor correlations with both parameters (R^2^ = 0.18 with intact cell concentration and R^2^ = 0.11 with intracellular ATP).
- Extracellular ATP constituted on average 36% of total ATP in the present study, which confirms the necessity of extracellular ATP subtraction from total ATP measurements during chlorinated drinking water analyses.
- Overall the results raise questions with respect to the offset between increased biological safety gained from disinfection opposed to increased risk from instability (uncontrolled bacterial growth). While an improvement of the chlorination procedure could be a solution, the data suggests looking beyond only disinfection for achieving biological stability of drinking water.

## Supporting Information

Figure S1 **Additional examples of hydrant flushing.** Changes in intact cell concentration and intracellular ATP during flushing in 6 newly-opened fire hydrants. Intact cell concentration values are shown as solid lines with blue markers, whereas intracellular ATP results displayed as single green bullets.
(TIF)

Figure S2 **Actual data for** **Figure 3A** **.** Changes in various bacterial parameters between one water treatment plant and a randomly selected point in the distribution network (actual values for Figure 3A).
(TIF)

Figure S3 **Residual chlorine concentration in the distribution network.** 50% of residual chlorine concentration in the network was between 0.12 (first quartile) and 0.23 (third quartile) mg mL^−1^, with a mean value of 0.17 mg mL^−1^ (n = 27). The whiskers indicate on minimum and maximum values, whereas bullets show outliers of the population.
(TIF)

Table S1 **Physical and chemical parameters of water measured on-line during low velocity flushing of newly-opened fire hydrant.** Some measurements were omitted during the first 20 minutes of flushing due to the high fluctuation in measuring tools readings.
(DOC)
