# Supplementary figures and images for: Biological Instability in a Chlorinated Drinking Water Distribution Network
Source: PLoS One. 2014 May 5;9(5):e96354. doi: 10.1371/journal.pone.0096354 (PMC4010465; doi:10.1371/journal.pone.0096354)

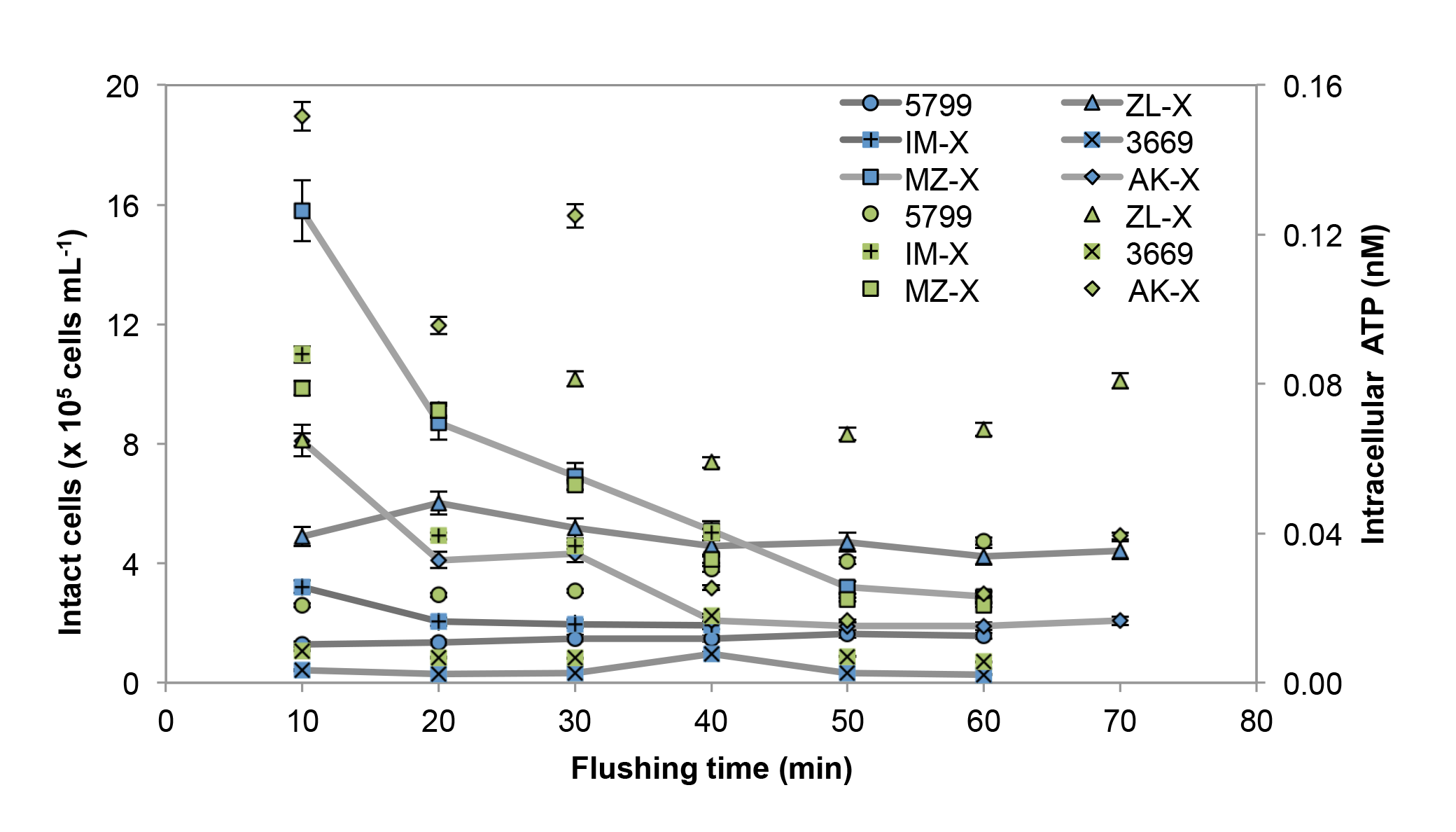

Supplement: Figure S1 — Additional examples of hydrant flushing. Changes in intact cell concentration and intracellular ATP during flushing in 6 newly-opened fire hydrants. Intact cell concentration values are shown as solid lines with blue markers, whereas intracellular ATP results displayed as single green bullets. (TIF) [file pone.0096354.s001.tif]

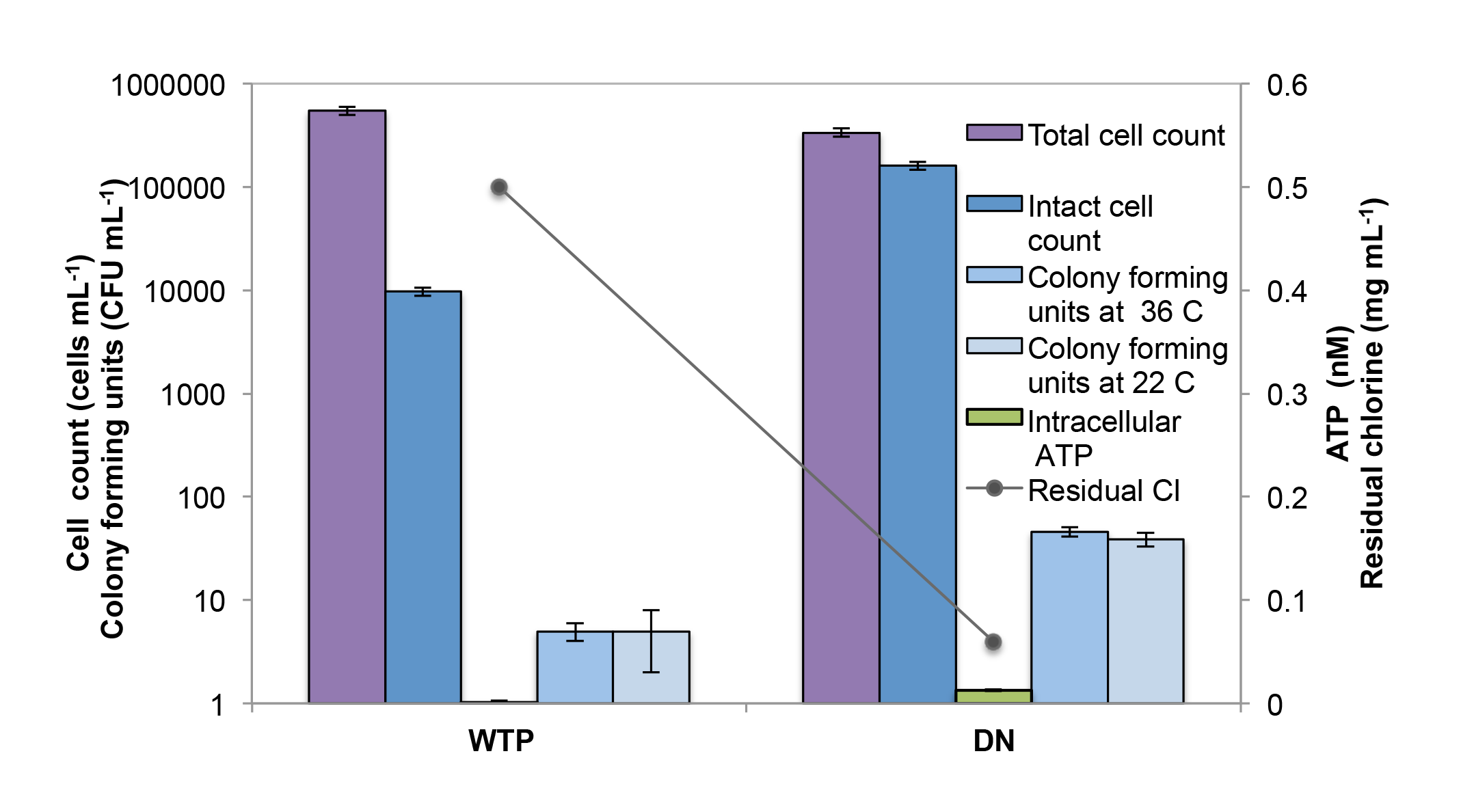

Supplement: Figure S2 — Actual data for Figure 3A . Changes in various bacterial parameters between one water treatment plant and a randomly selected point in the distribution network (actual values for Figure 3A). (TIF) [file pone.0096354.s002.tif]

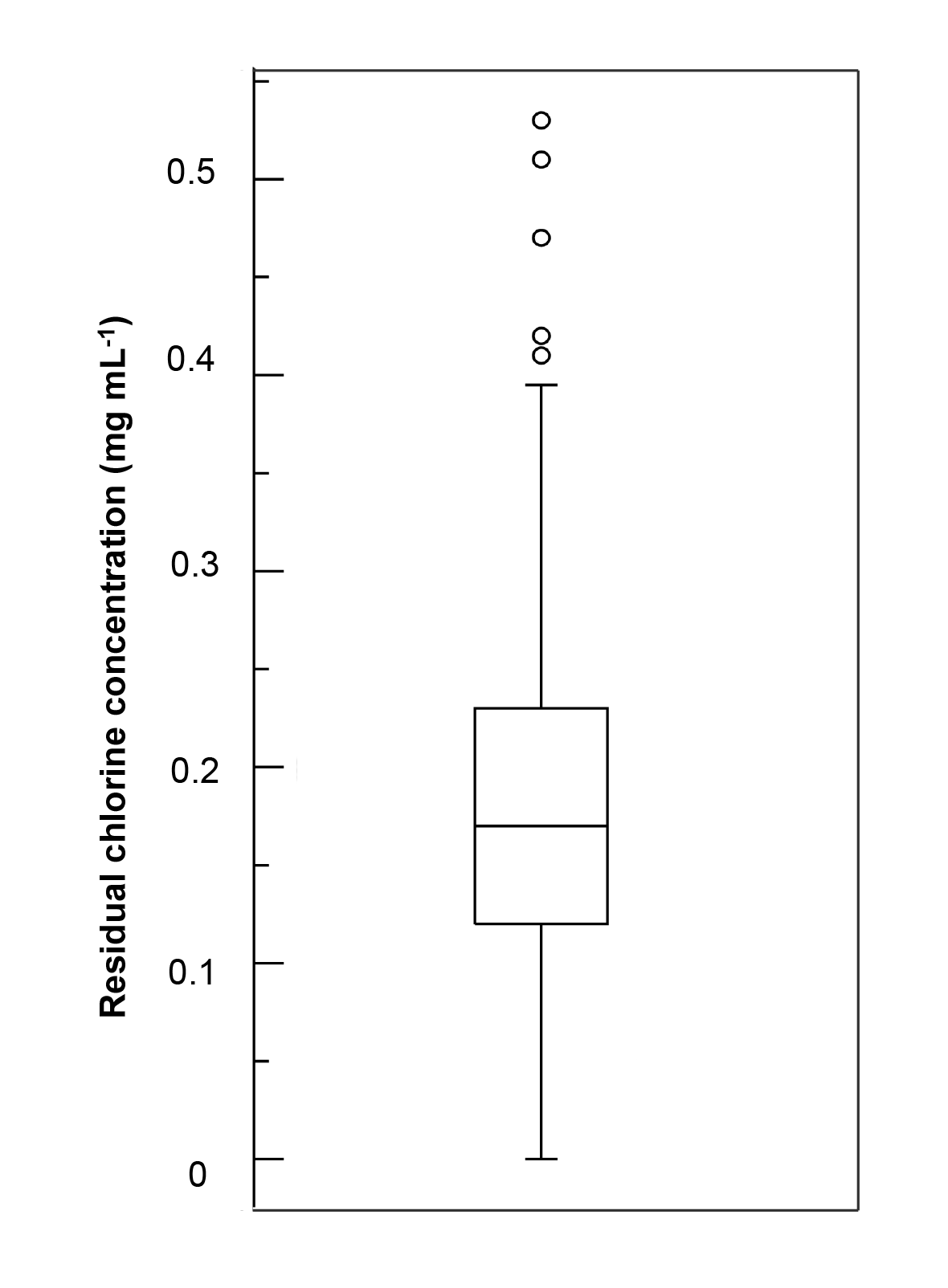

Supplement: Figure S3 — Residual chlorine concentration in the distribution network. 50% of residual chlorine concentration in the network was between 0.12 (first quartile) and 0.23 (third quartile) mg mL−1, with a mean value of 0.17 mg mL−1 (n = 27). The whiskers indicate on minimum and maximum values, whereas bullets show outliers of the population. (TIF) [file pone.0096354.s003.tif]
