# Supplementary material for: Biological Instability in a Chlorinated Drinking Water Distribution Network
Source: PLoS One. 2014 May 5;9(5):e96354. doi: 10.1371/journal.pone.0096354 (PMC4010465; doi:10.1371/journal.pone.0096354)
Supplement: Table S1 — Physical and chemical parameters of water measured on-line during low velocity flushing of newly-opened fire hydrant. Some measurements were omitted during the first 20 minutes of flushing due to the high fluctuation in measuring tools readings. (DOC) [file pone.0096354.s004.doc]

**Table S1.** **Physical and chemical parameters of water measured on-line during low velocity flushing of newly-opened fire hydrant.**

Some measurements were omitted during the first 20 minutes of flushing due to the high fluctuation in measuring tools readings.

| **Flushing time (min)** | **pH** | **Redox (mV H2)** | **EC (µS/cm)** | **Turbidity (FTU eq.)** | **Temperature (°C)** |
| --- | --- | --- | --- | --- | --- |
| 5 | - | - | - | 128.84 | - |
| 10 | 7.73 | 7 | 484 | 18.613 | 15.5 |
| 15 | - | - | - | 142.499 | - |
| 20 | - | - | - | 9.714 | - |
| 25 | 7.88 | 94 | 525 | 8.547 | 15.1 |
| 30 | 7.81 | 85 | 524 | 6.349 | 15 |
| 35 | 7.77 | 87 | 528 | 4.234 | 14.9 |
| 40 | 7.77 | 82 | 530 | 2.764 | 14.9 |
| 45 | 7.76 | 78 | 533 | 1.923 | 14.9 |
| 50 | 7.77 | 73 | 535 | 1.772 | 14.9 |
| 55 | 7.76 | 77 | 530 | - | 14.8 |
| 60 | 7.75 | 78 | 530 | - | 14.9 |
